# Supplementary material for: Evaluation of HIV Transmission Clusters among Natives and Foreigners Living in Italy
Source: Viruses. 2020 Jul 23;12(8):791. doi: 10.3390/v12080791 (PMC7472346; doi:10.3390/v12080791)
Supplement: Supplementary file 1 [file viruses-12-00791-s001.pdf]

viruses

MDPI

Supplementary Materials: Evaluation of HIV Transmission Clusters Among Natives and Foreigners Living in Italy

Lavinia Fabeni, Maria Mercedes Santoro, Patrizia Lorenzini, Stefano Rusconi, Nicola Gianotti, Andrea Costantini, Loredana Sarmati, Andrea Antinori, Francesca Ceccherini-Silberstein, Antonella d'Arminio Monforte, Annalisa Saracino, Enrico Girardi

Table S1. Characteristics of foreign individuals with HIV-1 living in Italy.

Variables	Overall	Out of cluster	In cluster	*p*-value[a]
	n = 695	613 (88.2%)	82 (11.8%)	
**Male gender, *n* (%)**	457 (65.8%)	383 (62.5%)	74 (90.2%)	<0.001
**Age, years, median (IQR)**	34 (28–40)	34 (28–40)	29 (26–37)	0.008
**Mode of HIV transmission, *n* (%)**				
F heterosexual	209 (30.0%)	201 (32.8%)	8 (9.8%)	<0.001
F IVDU	9 (1.3%)	9 (1.5%)	0 (0.0%)	
M heterosexual	134 (19.3%)	125 (20.4%)	9 (11.0%)	
M IVDU	25 (3.6%)	23 (3.8%)	2 (2.4%)	
MSM	243 (35.0%)	187 (30.4%)	56 (68.3%)	
Other/unknown	75 (10.8%)	68 (11.1%)	7 (8.5%)	
**Nation of birth, *n* (%)**				
Africa	219 (31.5%)	212 (34.6%)	7 (8.5%)	<0.001
Central and South America	241 (34.7%)	201 (32.8%)	40 (48.8%)	
Europe	187 (26.9%)	159 (25.9%)	28 (34.2%)	
Asia	38 (5.5%)	35 (5.7%)	3 (3.7%)	
Other	10 (1.4%)	6 (1.0%)	4 (4.8%)	
**Education, *n* (%)**				
**Years in Italy, median (IQR)**	5 (1–9)	5 (1–10)	7 (3–9)	0.488
Primary school	179 (11.4%)	73 (11.9%)	6 (7.3%)	0.236
Secondary school	126 (18.1%)	114 (18.6%)	12 (14.6%)	
College/University	223 (32.1%)	188 (30.7%)	35 (42.7%)	
Unknown	267 (38.4%)	238 (38.8%)	29 (35.4%)	
**Employment, *n* (%)**				
Employed	217 (31.2%)	188 (30.7%)	29 (35.4%)	0.206
Unemployed	176 (25.3%)	158 (25.8%)	18 (22.0%)	
Self-employed	73 (10.4%)	61 (9.9%)	12 (14.6)	
Student	15 (2.2%)	11 (1.8%)	4 (4.9%)	
Housewife	29 (4.2%)	28 (4.5%)	1 (1.2%)	
Other	74 (10.7%)	66 (10.8%)	8 (9.8%)	
Unknown	111 (16.0%)	101 (16.5%)	10 (12.1%)	
**HIV RNA, copies/mL, *n* (%)**				
<1000	38 (5.5%)	32 (5.2%)	6 (7.3%)	0.362
1000-10,000	145 (20.9%)	127 (20.7%)	18 (22.0%)	
10,000–100,000	257 (37.0%)	221 (36.1%)	36 (43.9%)	
>100,000	213 (30.6%)	195 (31.8%)	18 (22.0%)	
Unknown	42 (6.0%)	38 (6.2%)	4 (4.9%)	
**CD4, cells/mm^3, *n* (%)**				
≤200	195 (28.0%)	188 (30.7%)	7 (8.5%)	<0.001
201–500	285 (41.0%)	249 (40.6%)	36 (43.9%)	

>500	179 (25.8%)	145 (23.7%)	34 (41.5%)	
Unknown	36 (5.2%)	31 (5.0%)	5 (6.1%)	
**Year of diagnosis, median (IQR)**	2012 (2009–2014)	2011 (2009–2014)	2012 (2010–2014)	0.057
**Subtype, *n* (%)**				
A1	43 (6.2%)	41 (6.7%)	2 (2.4%)	<0.001
B	337 (48.5%)	282 (46.0%)	55 (67.1%)	
C	68 (9.8%)	64 (10.4%)	4 (4.9%)	
CRF02_AG	74 (10.7%)	69 (11.3%)	5 (6.1%)	
CRF60_BC	5 (0.7%)	0 (0.0%)	5 (6.1%)	
F1	61 (8.8%)	54 (8.8%)	7 (8.5%)	
Other	107 (15.3%)	103 (16.8%)	4 (4.9%)	

[a]By Mann-Whitney test (for quantitative variables) and χ2 test or Fisher's exact test (for categorical variables), as appropriate. *p* values <0.05 were considered statistically significant and were reported in bold. F: female; IVDU: intravenous drug user; M: male; MSM: men who have sex with men.

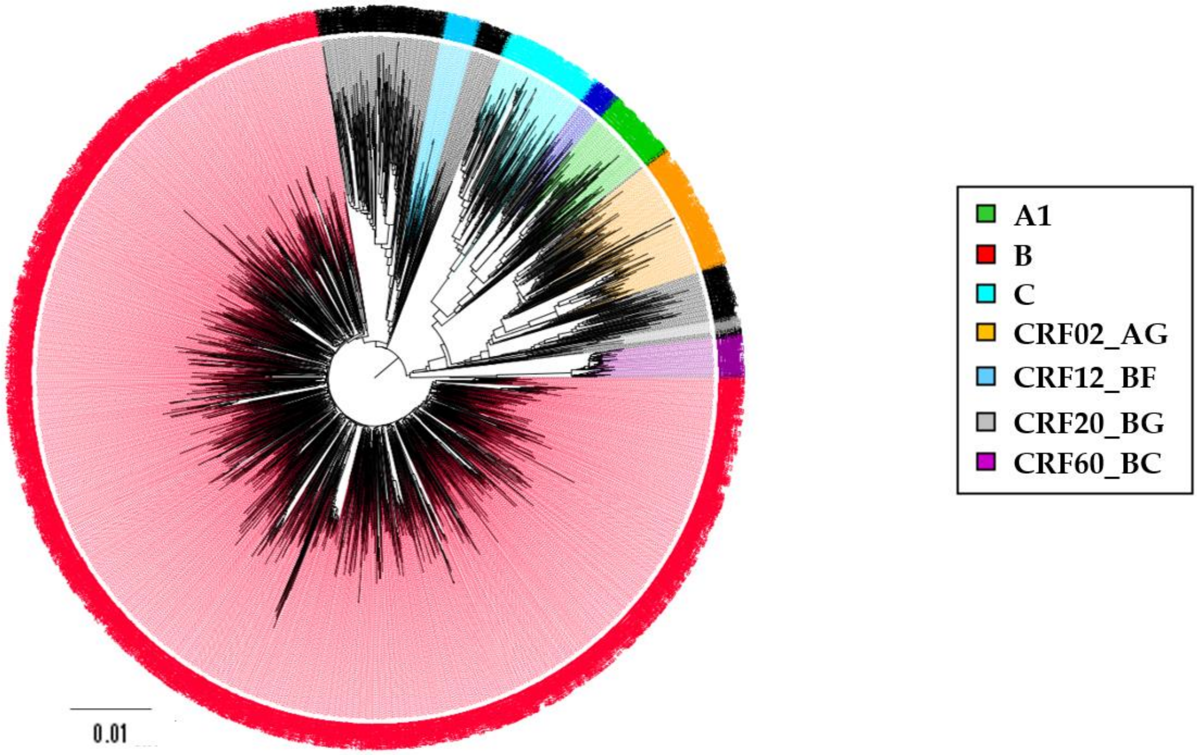

Figure S1. Phylogenetic tree of the 3499 *pol* sequences analysed and colored according to the most representative subtypes. Scale bar indicates estimated evolutionary distance of 0.01 substitution per position.

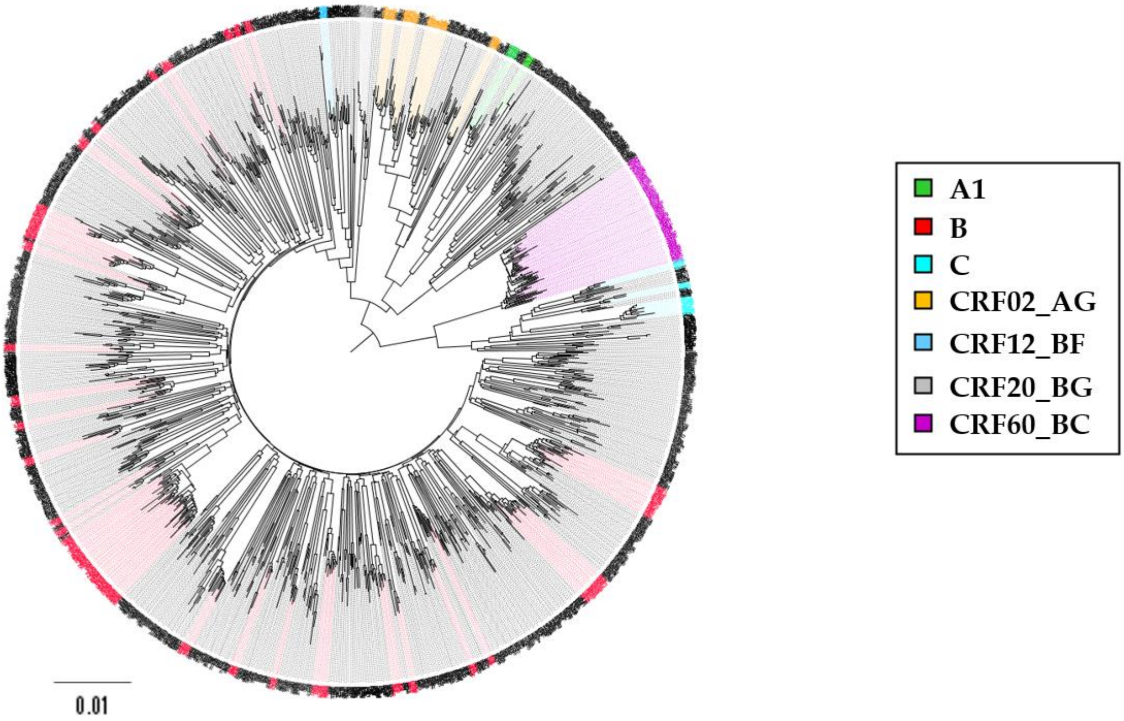

Figure S2. Phylogenetic tree of the 726 *pol* sequences involved in 228 molecular transmission clusters (MTCs). The 36 medium and the 6 large MTCs are colored according to HIV-1 subtype. The 186 small MTCs are shown in black. Scale bar indicates estimated evolutionary distance of 0.01 substitution per position.
